# Transcriptome-Based Modeling Reveals that Oxidative Stress Induces Modulation of the AtfA-Dependent Signaling Networks in *Aspergillus nidulans*

**DOI:** 10.1155/2017/6923849

**Published:** 2017-07-09

**Authors:** Erzsébet Orosz, Károly Antal, Zoltán Gazdag, Zsuzsa Szabó, Kap-Hoon Han, Jae-Hyuk Yu, István Pócsi, Tamás Emri

**Affiliations:** ^1^Department of Biotechnology and Microbiology, Faculty of Science and Technology, University of Debrecen, Debrecen, P.O. Box 63 H-4010, Hungary; ^2^Department of Zoology, Faculty of Sciences, Eszterházy Károly University, Eger, Eszterházy tér 1 H-3300, Hungary; ^3^Department of General and Environmental Microbiology, Faculty of Sciences, University of Pécs, Pécs, P. O. Box 266 H-7601, Hungary; ^4^Department of Pharmaceutical Engineering, Woosuk University, Wanju 565-701, Republic of Korea; ^5^Department of Bacteriology, University of Wisconsin, 1550 Linden Dr., Madison, WI 53706, USA

## Abstract

To better understand the molecular functions of the master stress-response regulator AtfA in *Aspergillus nidulans*, transcriptomic analyses of the *atfA* null mutant and the appropriate control strains exposed to menadione sodium bisulfite- (MSB-), *t*-butylhydroperoxide- and diamide-induced oxidative stresses were performed. Several elements of oxidative stress response were differentially expressed. Many of them, including the downregulation of the mitotic cell cycle, as the MSB stress-specific upregulation of FeS cluster assembly and the MSB stress-specific downregulation of nitrate reduction, tricarboxylic acid cycle, and ER to Golgi vesicle-mediated transport, showed AtfA dependence. To elucidate the potential global regulatory role of AtfA governing expression of a high number of genes with very versatile biological functions, we devised a model based on the comprehensive transcriptomic data. Our model suggests that an important function of AtfA is to modulate the transduction of stress signals. Although it may regulate directly only a limited number of genes, these include elements of the signaling network, for example, members of the two-component signal transduction systems. AtfA acts in a stress-specific manner, which may increase further the number and diversity of AtfA-dependent genes. Our model sheds light on the versatility of the physiological functions of AtfA and its orthologs in fungi.

## 1. Introduction

Oxidative stress is commonly defined as a physiological state when the negative effects of reactive oxygen species (ROS) significantly decrease the fitness of stress-exposed cells. Besides its practical importance, for example, oxidative stress occurs frequently during host-pathogen interactions, decomposition of xenobiotics, and biosorption of heavy metals [1–4], oxidative stress response is frequently studied in fungal biology to understand how these microbes are able to adapt to their rapidly changing environment and, in more general, to elucidate the molecular mechanisms of stress signalings and regulations [5–7]. Several events have been identified in the course of oxidative stress response so far, and many of them, including the activation of glutaredoxin-thioredoxin and DNA repair systems, production of antioxidant enzymes and NADPH, or inhibition of cell cycle, are observed commonly in a wide spectrum of species [4, 8–11].

AtfA and its orthologues (e.g., Atf1 in the fission yeast *Schizosaccharomyces pombe* or Atf2 in mammals) are conserved bZIP oxidative stress response elements regulated by MAPK (mitogen-activated protein kinase) pathways in eukaryotes [12, 13]. In *S. pombe*, Atf1 forms a heterodimer with Pcr1 and this heterodimer participates in meiotic recombination, maintenance of heterochromatin structure, and regulation of certain genes related to sexual differentiation, besides induction of stress-responsive genes under oxidative, heat, reductive, osmotic, and starvation stresses [13–17]. AtfA has been characterized as a regulator of conidial stress tolerance in *A. nidulans*, *A. fumigatus*, and *A. oryzae* [18–21]. As an example, more than half of the conidia-specific genes is regulated in an AtfA-dependent manner in *A. fumigatus*; among them, upregulation of conidial stress-related genes and downregulation of genes related to germination are notable [20]. AtfA regulates several processes in vegetative hyphae in filamentous fungi. It contributes to stress tolerance and/or alters secondary metabolism in *A. nidulans* [12, 22–24], *Fusarium graminearum* [25], *Fusarium oxysporum* [26], *Botrythis cinerea* [27], *Magnaporthe oryzae* [28], and *Claviceps purpurea* [29]. AtfA is an important component of a central multiple-stress signaling pathway also regulating development in filamentous fungi as well [24]. AtfB, an orthologue/paralogue of AtfA, is an important transcription factor which integrates mycotoxin production and oxidative stress response in *Aspergillus parasiticus* and probably in other aspergilli as well [30]. Most recently, the involvement of the bZIP-type transcription factors AtfA-D in the orchestration of stress responses mounted against various types of environmental stress was also demonstrated in *A. fumigatus* [31]. AtfA also influences asexual and/or sexual development in *Neurospora crassa*, *A. nidulans*, and *F. graminearum* [12, 24, 25, 32]. Owing to its importance in the regulation of stress tolerance and secondary metabolism, AtfA significantly contributes to the virulence of plant pathogenic fungi [25, 27–29], and it is also essential for the virulence of the human pathogenic *A. fumigatus* [19, 20, 31]. The involvement of AtfA in virulence and/or mycotoxin production in several fungi explains the ceaseless interest in this bZIP-type transcription factor.

In a previous study, we investigated the genome-wide transcriptional changes mounted in *A. nidulans*, when it was exposed to six types of stresses, including oxidative stress (menadione sodium bisulfite (MSB), low and high concentration of H_2_O_2_, *t*-butylhydroperoxide (tBOOH), diamide) and high-osmolarity stress (NaCl) [23]. Transcriptional changes taking place in stress-exposed vegetative tissues of exponentially growing *A. nidulans* were recorded and compared in an oxidative stress-sensitive *ΔatfA* mutant as well as in the appropriate control strains [23]. The observed stress responses were quite different at the level of the stress responsive genes, which was unexpected since out of the six studied stress conditions, five were oxidative stresses [23]. In this study, we carried out a functional categorization of the stress-responsive genes to identify gene groups and biological processes which were under AtfA control in oxidative stress-treated vegetative hyphae. To reach these goals, three stress treatments (MSB, tBOOH, and diamide) were chosen and studied in details because the selected oxidative stress conditions were similar to each other in strength, according to the high and comparable numbers of stress-responsive genes and the significant and also comparable growth inhibitions observed in stress-exposed cultures [23]. As a result, several AtfA-dependent elements and cellular events of oxidative stress response were identified based on stress-elicited transcriptional changes, including the downregulation of mitotic cell cycle genes, nitrate reduction, tricarboxylic acid cycle, and ER to Golgi vesicle-mediated transport or the upregulation of FeS cluster assembly genes. To elucidate how AtfA is able to regulate these versatile biological processes, we set up a model based on transcriptomic data which suggests that the main function of AtfA is to modulate the signaling network operating under oxidative stress.

## 2. Materials and Methods

### 2.1. Strains and Culture Conditions

The *A. nidulans* TNJ 92.4 (*pyrG89*, *AfupyrG*^+^; *pyroA4*; *ΔatfA:pyroA*; *veA*^+^) and THS30.3 (*pyrG89*, *AfupyrG*^+^; *pyroA*^+^; *veA*^+^) strains as a *ΔatfA* gene deletion mutant and the appropriate control strain, respectively [23], were used in this study. The strains were maintained on Barratt's minimal medium [33], and the inoculated agar plates were incubated at 37°C for 6 d. Conidia harvested from these cultures were used to inoculate submerged liquid cultures. All liquid cultivations were carried out in Erlenmeyer flasks (500 ml) containing 100 ml Barratt's minimal medium, inoculated with 1 × 10^8^ conidia and incubated at 37°C and at 3.7 Hz shaking frequency for 20 h [23]. Stress exposures were carried out at 16 h using cultures with similar biomass concentrations as described earlier [23]. The applied stressor concentrations (0.12 mM MSB, 0.8 mM tBOOH, and 1.8 mM diamide) were close to those used by other researchers earlier [12, 18] and reduced (but did not block completely) the growth of both strains with similar intensity [23]. Samples were taken at 0.5 h for RNA isolation and at 4 h for measuring specific enzyme activities, sterol contents, and extracellular siderophore contents after stress exposures. Independent cultures were used for microarray experiments, RT-qPCR tests, and for physiological characterizations.

### 2.2. Reverse Transcription Quantitative Real-Time Polymerase Chain Reaction (RT-qPCR) Assays

**Figure 1:**
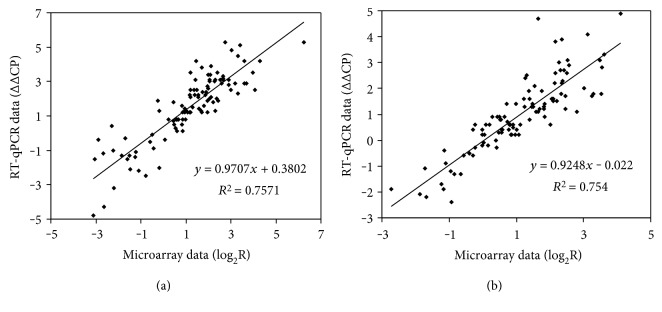
Correlation between microarray and RT-qPCR data in case of the control (a) and the *ΔatfA* (b) strains.

Total RNA was isolated from lyophilized mycelia according to Chomczynski [34] and RT-qPCR experiments were carried out as described earlier [23]. The applied primer pairs are presented in Supplementary Table 1 available online at https://doi.org/10.1155/2017/6923849. Relative transcription levels were quantified with the ΔΔCP value (mean ± S.D. calculated from 4 biological replicates). ΔΔCP was defined as ΔCP_treated_ − ΔCP_control_, where ΔCP_treated_ = CP_reference gene_ − CP_tested gene_ measured in stress-treated cultures, ΔCP_control_ = CP_reference gene_ − CP_tested gene_ measured in untreated cultures, and CP values stand for the RT-qPCR cycle numbers of crossing points. As reference gene, *actA* (AN6542) was used [35]. RT-qPCR experiments were carried out in the Genomic Medicine and Bioinformatics Core Facility, University of Debrecen, Debrecen, Hungary. RT-qPCR data showed strong correlation with microarray data in both strains (Figures 1(a) and 1(b)).

### 2.3. Enzyme Activity Assays

Specific enzyme activities were determined from cell-free extracts prepared by X-pressing [36] according to the protocol of Chiu et al. [37] (glutathione peroxidase (GPx)), Pinto et al. [38] (glutathione reductase (GR)), Roggenkamp et al. [39] (catalase), Emri et al. [36] (glucose-6-phosphate dehydrogenase (G6PD)), and Bruinenberg et al. [40] (nitrate reductase (NR)). Protein content of the samples was determined with Bradford reagent [41].

### 2.4. Sterol Content Determination

Total sterol measurement was performed according to Arthington-Skaggs et al. [42] using lyophilized mycelia. Samples were saponificated with 25 *w*/*v* % KOH dissolved in 65 *v*/*v* % ethanol for 1 h at 85°C and sterols were extracted with *n*-heptane. The sterol content of the heptane phase was determined spectrophotometrically using a standard curve made with ergosterol. All samples were taken at 4 h after stress treatments.

### 2.5. Extracellular Siderophore Production

Siderophore content was determined as described earlier [43] using fermentation broths or concentrated (10×) fermentation broths prepared by lyophilization as samples.

### 2.6. Evaluation of the Microarray Data

Normalized DNA chip data (Gene Expression Omnibus; accession number GSE63019) were obtained from the experiments described earlier [23] using Agilent 60-mer oligonucleotide high-density arrays (4 × 44K; design number 031140; Kromat Ltd., Budapest, Hungary). Total RNA samples were isolated from lyophilized mycelia originated from untreated and stress-treated cultures. RNA samples gained from three independent experiments were pooled in 1 : 1 : 1 ratio and these mixtures were used for DNA chip experiments.

Genes represented by oligomer probes on the DNA chip but modified (splitted, merged) or deleted from the genome during the most recent revisions (AspGD; http://www.aspergillusgenome.org) were omitted from the evaluation, and the modified gene list was used in further analyses. Stress-responsive genes (genes upregulated or downregulated by the stress treatment) were selected by the D1 metric (a derivative of the J5 test [44, 45]) with threshold 3. Coregulated genes (core oxidative stress response genes; [23]) were defined as genes showing unidirectional stress-responsive behavior in all the three stresses applied. Uniquely regulated genes were defined as genes upregulated or downregulated only in one out of the three applied stresses. AtfA-dependent genes were regarded as genes where upregulation (downregulation) was detected in the control strain but no regulation or regulation on the opposite direction was observed in the *ΔatfA* mutant regardless of the SI_treated,control_/SI_treated,*ΔatfA*_ ratio (SI stands for the normalized microarray signal intensity).

Gene enrichment analysis was carried out with the AspGD Gene Ontology Term Finder (http://www.aspergillusgenome.org/cgi-bin/GO/goTermFinder) applying default settings, using the appropriate background gene set (i.e., the modified gene list of the DNA chip) and biological process ontology GO terms. The FungFun2 package (https://elbe.hki-jena.de/fungifun/fungifun.php), with default settings and the appropriate background gene set, was also used to test the enrichment of genes related to FunCat categories in selected gene groups [46]. Only hits with a *p* value of <0.05 were taken into consideration during the evaluation process.

In addition to the sets of stress-responsive, uniquely regulated, coregulated, and atfA-dependent genes, groups of functionally related genes were also generated and studied by extracting information from the *Aspergillus* Genome Database (http://www.aspergillusgenome.org) unless otherwise indicated. Typically, these gene groups contain all genes described by the mentioned GO terms or by their child terms. The following gene groups were generated and used in the further evaluation of the transcriptomic data:

- “Ribosome biogenesis,” “mitotic cell cycle,” “iron-sulfur cluster assembly,” and “ER to Golgi vesicle-mediated transport” genes.
- “Two-component signal transduction system” genes. These groups contain all genes directly related to these FunCat terms according to the FungiFun2 server (https://elbe.hki-jena.de/fungifun/fungifun.php).
- “Antioxidant enzyme” genes. This gene group includes genes encoding known and likely antioxidant enzymes (*Aspergillus* Genome Database; [47]).
- “Siderophore biosynthesis” genes. This group of genes contains all genes directly involved in the “siderophore biosynthetic process”, “positive regulation of siderophore biosynthetic process” and in the “N′,N″,N‴-triacetylfusarinine C biosynthetic process.”
- “Nitrate utilization” genes. This group contains all genes directly related to the “nitrate transmembrane transporter activity,” “nitrite uptake transmembrane transporter activity,” “nitrate reductase (NADPH) activity,” “nitrite reductase (NAD(P)H) activity,” “nitrate assimilation,” and “regulation of nitrate assimilation.”
- “Squalene-ergosterol pathway” genes. This gene group contains the orthologues of *A. fumigatus* genes [48] encoding enzymes involved in ergosterol biosynthesis from squalene.
- “Signal transduction” genes. This group contained solely those stress-responsive genes, which belonged to the “signal transduction” GO term or to its child terms.

## 3. Results

### 3.1. Genome-Wide Transcriptional Changes Caused by atfA Deletion

**Figure 2:**
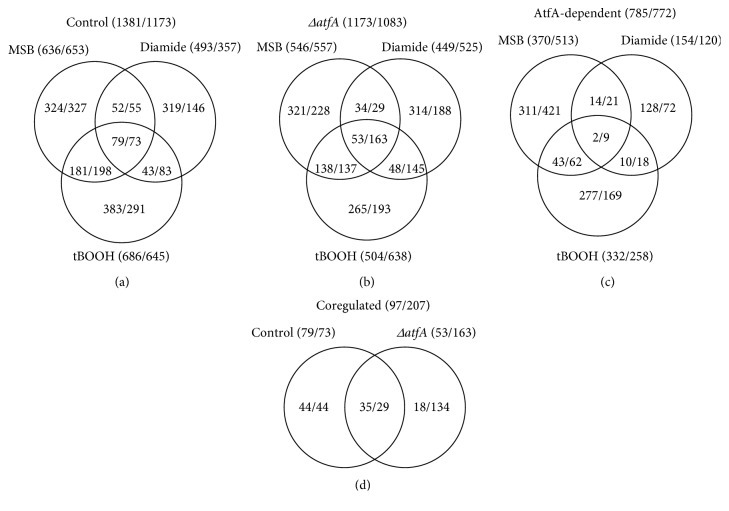
Venn-diagram of stress-responsive genes. (a) Distribution of stress-responsive (upregulated/downregulated) genes among the 3 oxidative stresses in the control strain. (b) Distribution of stress-responsive (upregulated/downregulated) genes among the 3 oxidative stresses in the *ΔatfA* strain. (c) Distribution of AtfA-dependent genes (showing upregulation/downregulation in the control strain) according to their stress dependence lost in the mutant strain. (d) Distribution of coregulated genes between the two strains. Stress-responsive, upregulated, downregulated, AtfA-dependent, and coregulated genes are defined in Materials and Methods.

Global transcriptional changes in *A. nidulans* under MSB, tBOOH, and diamide exposures were detected and compared (Figures 2(a) and 2(b)). Altogether, the upregulation of 785 genes as well as the downregulation of 772 genes showed AtfA dependence in at least one stress condition in our experiments (Figure 2(c)). The most AtfA-dependent genes were found among the MSB stress-dependent genes: out of the 1557 aforementioned genes, 883 (57%) were AtfA dependent under MSB stress (Figure 2(c)). The majority of the AtfA-dependent genes showed AtfA dependence only under one stress treatment (Figure 2(c)): only 11 genes (0.7%) showed AtfA dependence under all the three stress conditions tested. It also meant that AtfA affected the transcription of different genes under different stress conditions (Figure 2(c)).

The numbers of coregulated genes (which were regarded as core oxidative stress response genes earlier [23]) were 79 + 73 = 152 and 53 + 163 = 216 in the control and the *ΔatfA* strains, respectively (Figures 2(a) and 2(b)), which numbers represent only 6–10% of the stress-responsive genes. Deletion of *atfA* did not decrease the total number of coregulated stress-responsive genes and the overlap between the two coregulated gene groups was relatively small (Figures 2(a), 2(b), and 2(d)). In other words, deletion of *atfA* not only prevented the coregulation of genes (altogether 88 genes) but also resulted in a number of new coregulations (altogether 152 genes) (Figure 2(d)).

Changes in the regulation of AtfA-dependent genes elicited by deleting the *atfA* gene itself are summarized in Supplementary Table 2. These data provided us with the following pieces of information: (i) Lots of genes (1045 genes) lost their stress responsiveness in the *ΔatfA* mutant, while lots of other genes (704 genes) became stress responsive in this strain. (ii) Many coregulated genes (88 genes) lost their coregulated nature while others (152 genes) became coregulated. (iii) Lots of tBOOH stress-specific genes (altogether 312 genes) gained MSB stress dependence in the *ΔatfA* strain. Although the deletion of *atfA* elicited further changes in the stress responsiveness of other stress-dependent genes as well, the numbers of affected genes were typically much lower and varied only between 52 (diamide-dependent genes, which gained tBOOH dependence) and 120 (tBOOH-dependent genes, which gained diamide dependence), as a function of the actual oxidative stress treatments employed (Supplementary Table 2).

### 3.2. Functional Categorization of Stress-Responsive Genes

Gene-enrichment analysis of stress-responsive genes resulted in several significant shared GO and FunCat terms which are presented in Supplementary Table 3 and a list of selected terms is shown in Supplementary Table 4. Gene enrichment analysis of the AtfA-dependent genes resulted in several very different biological process terms which are not related tightly to oxidative stress response (Table 1, Supplementary Table 3).

### 3.3. AtfA Dependence of Selected Gene Groups

AtfA-dependent and AtfA-independent regulations of 10 gene groups were traversed by us and our findings are presented here in details.

#### 3.3.1. “Ribosome Biogenesis” Genes

**Figure 3:**
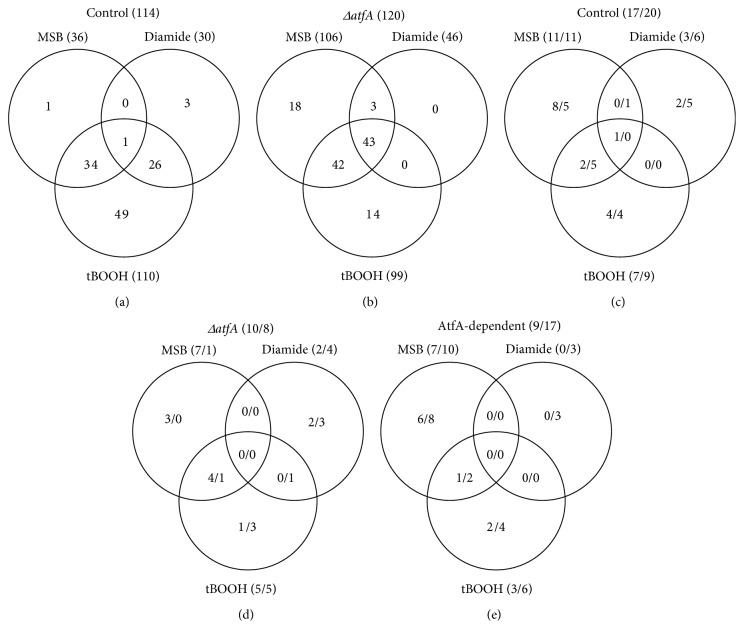
Stress-type dependence of “ribosome biogenesis” and “signal transduction” genes. (a and b) Distribution of downregulated “ribosome biogenesis” genes among the 3 stresses in the control and the *ΔatfA* strain, respectively. (c and d) Distribution of upregulated/downregulated “signal transduction” genes among the 3 stresses in the control and the *ΔatfA* strains, respectively. (e) Distribution of AtfA-dependent “signal transduction” genes (showing upregulation/downregulation in the control strain) according to their stress dependence lost in the mutant strain.

“Ribosome biogenesis” genes were significantly enriched under all three stresses in both the control and the *ΔatfA* mutant strains when downregulated genes were analyzed (Supplementary Table 5). Interestingly, different genes were downregulated under MSB than under diamide stress and therefore, the number of coregulated genes was low (1 gene). Deletion of *atfA* significantly increased the number of downregulated genes under MSB stress (Supplementary Table 5). Several genes which were tBOOH or tBOOH-diamide stress dependent became MSB stress dependent as well and as a consequence of the number of coregulated genes increased from 1 to 43 genes (Figures 3(a) and 3(b)).

#### 3.3.2. “Mitotic Cell Cycle” Genes

Downregulated “mitotic cell cycle” genes were significantly enriched in all three stress treatments in the control strain (Supplementary Table 5). Deletion of *atfA* significantly decreased the number of downregulated genes under MSB, tBOOH, and diamide stresses (Supplementary Table 5).

#### 3.3.3. Genes Encoding Antioxidant Enzymes

“Antioxidant enzyme” genes were significantly enriched in both strains in all three stress exposures when upregulated genes were studied (Supplementary Table 5). Deletion of *atfA* had only minor effects on the transcription of these genes (Supplementary Table 5). These upregulations were confirmed in both strains by RT-qPCR in case of several genes (Table 2). Moreover, elevated specific GR, GPx, and catalase activities were measured in both strains after stress treatments (Table 3).

#### 3.3.4. Genes Involved in Siderophore Biosynthesis

“Siderophore biosynthesis” genes were significantly enriched in the upregulated tBOOH stress-dependent gene groups of both the control and the *ΔatfA* mutant strains (Supplementary Table 5). The deletion of neither *atfA* nor MSB and diamide treatments had significant effects on this gene group (Supplementary Table 5). Upregulation of *hapX* and *sidA* under tBOOH stress in both strains was also supported by RT-qPCR data and these two genes showed upregulation under MSB stress as well in the control strain (Table 2). Interestingly, extracellular siderophore accumulations were not detected in any of the cultures (data not shown).

#### 3.3.5. “Iron-Sulfur Cluster Assembly” Genes

The upregulated “iron-sulfur cluster assembly” genes were significantly enriched under MSB stress in the control strains (Supplementary Table 5). Deletion of *atfA* significantly decreased the number of upregulated “iron-sulfur cluster assembly” genes from 9 to 4 under MSB stress (Supplementary Table 5). The behavior of these genes (AtfA-dependent regulation under MSB stress) was justified by testing the transcription of selected 11 genes with RT-qPCR: All the 11 genes were upregulated under MSB stress in the control strain but only 5 of them showed upregulation in the *ΔatfA* mutant (Table 2). In case of tBOOH and diamide stresses, 11 and 9 genes showed upregulation in the control strain, respectively, and 8 genes had elevated mRNA level in the mutant strain under both stress conditions (Table 2).

#### 3.3.6. “Two-Component Signal Transduction System” Genes

The enrichment of the upregulated “two-component signal transduction system” genes was significant only under MSB stress in the control strain, and deletion of *atfA* decreased the number of upregulated genes from 4 to zero (Supplementary Table 5). The AtfA-dependent upregulation of these genes under MSB stress treatment was also demonstrated in RT-qPCR measurements: all the tested 7 genes were upregulated under MSB stress in the control strain and only 1 of them showed elevated transcription in the mutant (Table 2).

#### 3.3.7. Nitrate Utilization Genes

The enrichment of these genes (altogether 14 genes) was significant only in case of the MSB stress (control strain) when the downregulated genes were studied (Supplementary Table 5) and deletion of *atfA* decreased the number of downregulated genes from 4 to zero. It is noteworthy that the cluster containing the genes, *niaD*, *niiA*, and *crnA* (encoding nitrate reductase, nitrite reductase, and nitrate/nitrite transporter, resp.; [49]) showed significantly reduced transcription in all three stress treatments in the control strain and this downregulation was clearly AtfA dependent in case of MSB treatment according to the RT-qPCR measurements (Table 2). Moreover, significantly reduced nitrate reductase activities were detected in all stress treatments but not under MSB stress in the *ΔatfA* strain (Table 3).

#### 3.3.8. “ER to Golgi Vesicle-Mediated Transport” Genes

“ER to Golgi vesicle-mediated transport” genes were significantly enriched under MSB stress in the control strain when downregulated genes were analyzed (Supplementary Table 5). Deletion of *atfA* significantly decreased the number of downregulated genes from 12 to 4 under MSB stress (Supplementary Table 5).

#### 3.3.9. Squalane-Ergosterol Biosynthetic Pathway Genes

Although a few genes showed downregulation under stress treatments, their enrichment was not significant in any case (Supplementary Table 5). Sterol measurement demonstrated that the sterol content was significantly decreased in the tBOOH-treated cultures of both strains (Supplementary Table 6).

#### 3.3.10. “Signal Transduction” Genes

Many signal-transduction-related genes (37 and 18 in the control and the mutant strains, resp.) were stress responsive in our experiments (Supplementary Table 5), and most of them were uniquely regulated under one stress condition (Figures 3(c) and 3(d), Supplementary Table 5). Besides the upregulation of *tcsA*, *hk2*, *hk-8-2*, and *hk-8-3*, two-component signal transduction system genes, which was characteristic for MSB-treated control cultures, the upregulations of *pdeA* (coding for a low-affinity cAMP phosphodiesterase [50]) and *lreB* (encoding a protein involved in blue-light-responsive differentiation and secondary metabolite production [51]) were also observed in diamide-treated *ΔatfA* and control cultures. In addition, the upregulations of *hsp90* heat shock protein and AN4419 (putatively encoding a tyrosine phosphatase) were detected typically under tBOOH stress (Supplementary Table 5). Deletion of *atfA* significantly decreased the number of downregulated signal transduction genes from 11 to 1 under MSB stress (Supplementary Table 5). The majority of the 26 AtfA-dependent signal-transduction-related genes (17 genes) lost their MSB stress dependence in the *ΔatfA* deletion strain (Figure 3(e)).

## 4. Discussion

In a previous study, we generated an *A. nidulans ΔatfA* mutant and the appropriate control strain [23]. The mutant strain showed elevated oxidative stress sensitivity on surface cultures in the presence of MSB, tBOOH, diamide, and H_2_O_2_ [23]. To gain information on the physiological changes in *A. nidulans* under oxidative stress as well as on the role of AtfA in the regulation of oxidative stress response, DNA chip experiments were conducted using submerged liquid cultures. According to these data, the stress responses were unexpectedly different in each oxidative stress treatment in both strains, which were characterized with few coregulated and high number of uniquely regulated genes. Moreover, the number of coregulated genes sharply decreased when the number of studied stress-initiating agents was increased, suggesting that the existence of a *Saccharomyces cerevisiae*-type (general) environmental stress response is very unlikely in *A. nidulans* [23]. Deletion of *atfA* affected mRNA accumulation of an unexpectedly high number of genes after MSB exposure, but the transcription of several genes showed AtfA dependence under the other stress conditions and even in untreated cultures [23]. Further analysis of stress-responsive genes detected under MSB, tBOOH, and diamide stresses in the control strain and in a *ΔatfA* mutant strain was carried out in order (i) to understand why the oxidative stress responses were very different at the level of transcriptome, (ii) to identify the gene groups/biological processes, which are under the control of AtfA in oxidative stress-exposed cultures, and (iii) transcriptome data were also used to set up hypotheses describing how AtfA contributes to the regulation of these gene groups.

### 4.1. Oxidative Stress Response Elements Revealed by Transcriptomic Data

Oxidative stress inhibited both the mitotic cell cycle and ribosome biogenesis (mRNA translation) in all three stress treatments studied (Supplementary Table 5). Their inhibition is a typical element of stress responses under strong stresses in fungi [10, 11, 52]. Stress-exposed cells can save lots of energy and materials in this way, which can be used to cope with the stress condition itself, and moreover, it prevents damages or even cell death caused by improper translation of proteins or erroneous cell cycle [52].

Upregulation of genes encoding antioxidative enzymes is among the most typical and characteristic stress response steps under oxidative stress [8–11]. This phenomenon was also observed in all three stress treatments we employed (Supplementary Table 5, Tables 2 and 3).

Although the efficient activity of the thioredoxine, glutaredoxine, and glutathione systems needs a high-level and continuous supplementation of NADPH [53], no upregulation of oxidative pentose phosphate shunt, which is one of the most important NADPH-producing pathways in fungi grown on glucose carbon source, was observed (Supplementary Table 3, Table 3). This observation is unexpected because the inductions of genes encoding G6PDH and 6-phosphogluconate dehydrogenase (6PGDH) are among the commonest oxidative stress response steps in yeasts [9, 52]. In addition, upregulation of GsdA (G6PDH) was also observed in proteomic analysis of long-term MSB-treated *A. nidulans* cultures [54] meanwhile no elevated specific G6PD and 6PGDH activities were detected in a high *β*-lactam producer *Penicillium chrysogenum* strain under oxidative stress [55, 56]. We can hypothesize that an increased flux of the oxidative pentose phosphate shunt may have been reached by regulatory mechanisms other than the transcriptional regulation of the genes encoding pathway-specific enzymes, for example, through decreasing the metabolite flux through the glycolytic pathway as it has been described in several organisms [57]. Alternatively, the upregulation of some other NADPH-producing processes, which were not identified in these experiments, for example, NADP isocitrate dehydrogenase [58] or the interconversion of glycerinaldehyde-3-phosphate and glycerol as described in yeasts [59], as well as the reduction of NADPH consumption dispensable in stress-exposed cultures, may also have provided stress-exposed *A. nidulans* cells with satisfactory quantities of NADPH to minimize the deleterious effects of oxidative stress exposures.

Regarding the NADPH-consuming processes, which are not directly coupled to oxidative stress defense, the nitrate reduction cluster (*niaD*, *niiA*, and *crnA*; [49]) was repressed under all stress conditions (Supplementary Table 5, Tables 2 and 3). The oxidative stress-dependent inactivation of nitrate reduction was also detected previously in *P. chrysogenum* [60]. It is reasonable to assume that the reduced metabolization of nitrate helps cells to provide them with enough NADPH to neutralize the deleterious effects of the oxidative stress-generating agents, but other explanations should also be considered. For example, the reduced metabolization of nitrate can also be a simple consequence of the reduced growth recorded in stress-exposed cultures [23] or can also prevent the formation of various harmful reactive nitrogen compounds, for example, nitric monoxide [61]. Importantly, the genes of nitric oxide-metabolizing proteins (*fhbA* and *fhbB*) are coregulated with the nitrate reduction cluster genes in this fungus [62].

Oxidative stress caused profound alterations in the primary metabolism as well. For example, the transcriptions of several genes related to both amino acid biosynthesis and degradation were altered (Supplementary Tables 3 and 4), which was likely the consequence of the cutback of de novo protein synthesis, which obviously perturbed the homeostasis of amino acids.

Although the aforementioned changes were observed in all three (in the case of amino acid metabolism, two of three) stresses, it did not mean that the upregulation or downregulation of these processes were necessarily ensured by an outstandingly high number of coregulated genes. The most characteristic example is the behavior of “ribosome biogenesis” genes: Out of the 110 downregulated genes, only one showed downregulation under all the three stress conditions studied; however, the number of downregulated genes was considerable in each individual stress treatment (Figure 3(a)). This observation is a good example of that; even if the overall changes in the stress response processes are similar to each other, the responses recorded at the level of the expression of individual stress genes may follow unique, stress-type-specific patterns. Not surprisingly, several biological processes were identified, which were characteristic for one stress condition only, which is also in line with the observed differences between the transcriptional changes detected at the level of individual genes.

Upregulation of peroxisome-related processes (“protein localization to peroxisome,” “peroxisomal transport,” and “fatty acid *β*-oxidation”) was observed only under tBOOH stress, which is in good accordance with the well-known lipid-damaging nature of this stressor [63]. A reduced production of sterols has been reported as a typical event of oxidative stress in order to maintain the fluidity of membranes under lipid peroxidation [64]. Although a few genes showed downregulation, enrichment of the downregulated ergosterol biosynthesis genes—in our case—was not detected under the studied stress conditions (Supplementary Table 5). However, the detected reduced sterol content of tBOOH-treated cells demonstrated that alterations in ergosterol synthesis can be an important oxidative stress response in *A. nidulans* even if these changes are not regulated or at least were not detectable at the level of transcriptome.

Upregulation of several “siderophore biosynthesis” genes was also characteristic for the tBOOH-induced oxidative stress response. Emerging data suggest that siderophores can have other physiological functions aside from iron uptake or storage. Peralta et al. demonstrated that enterobactin, a siderophore produced by *Escherichia coli*, protects cells from oxidative stress and this protection is independent of its iron-scavenging activity [65]. Moreover, it was also suggested that reduced siderophore content enhances oxidative stress sensitivity of *A. fumigatus* [66]. Unfortunately, we failed to detect siderophores from the fermentation broth of tBOOH-treated cultures at 4 h after stress treatment. Hence, further studies are needed to reveal the significance of the transcriptional changes observed with “siderophore biosynthesis” genes.

Downregulation of ER-specific processes under MSB stress (“protein localization to endoplasmic reticulum,” “ER to Golgi vesicle-mediated transport”) was also remarkable. Recent studies demonstrated that oxidative protein folding in ER and ER-associated NADPH oxidases are important sources of reactive oxygen species including superoxide [67, 68], and as a consequence, the downregulation of ER-related processes can be a relevant response to the elevated intracellular superoxide levels elicited by MSB. Upregulation of genes involved in FeS cluster assembly (Supplementary Table 5) was also among the foreseeable elements of MSB stress responses [69, 70], and it is in good accordance with the widely known sensitivity of the FeS cluster proteins to increasing intracellular superoxide levels [71]. Not surprisingly, downregulation of tricarboxylic acid cycle, which contains several FeS cluster proteins, was also observed specifically under MSB stress (Supplementary Table 5).

The observed stress-type-specific differences between the detected global transcriptional changes are in good accordance with the stress-type-dependent regulations of various signal transduction genes (Figure 3(c)). These data convincingly demonstrate that MSB, tBOOH, and diamide induced quite different stress responses in *A. nidulans*, and the differences observed either in the groups of stress-responsive genes or in the biological processes set into operation under various types of oxidative stress treatments cannot be explained merely with a few signaling pathways responding uniformly to each oxidative stress condition tested. Instead, the regulations of these pathways followed different patterns under different oxidative stress conditions. Although many of the abovementioned oxidative stress-dependent biological processes were under the control of AtfA, these AtfA-dependent regulations also showed high stress-type specificity. The AtfA-dependent biological processes include the downregulation of mitotic cell cycle (under all the three studied stress conditions), the MSB stress-specific upregulation of FeS cluster assembly, and the MSB stress-specific downregulation of nitrate reduction, tricarboxylic acid cycle, or ER to Golgi vesicle-mediated transport. The diversity of the AtfA-dependent biological processes together with the high number of AtfA-dependent downregulated genes (besides the upregulated ones) supports the view that the majority of the observed changes are only indirect consequences of *atfA* deletion. Interestingly, the upregulation of antioxidant enzymes did not show AtfA dependence; however, several studies have demonstrated the AtfA/Atf1-dependent induction of genes encoding catalases or GPx [22, 24, 72]. Most likely, the upregulation of these genes is under the control of several transcription factors which can substitute one and others under certain conditions.

### 4.2. The Possible Role of AtfA in the Regulation of Oxidative Stress Response

In order to elucidate how AtfA regulates oxidative stress response, we set up a hypothesis based on the following assumptions generated by transcriptomic data:

Assumption 1. AtfA regulates (directly and indirectly) many genes encoding elements of the stress signaling network. This assumption explains why *atfA* deletion affected a great number of genes with versatile functions and how AtfA can contribute to both the upregulation and downregulation of these genes.

Altogether, 26 genes encoding or putatively encoding signal transduction proteins showed AtfA-dependent expression in our experiments (Figure 3(e), Supplementary Table 5). Among them, some members of the “two-component signal transduction system” gene group (*tcsA*, *phkB*, *hk2*, *hk-8-2*, *hk-8-3*, and *hk-8-6*) (Table 2, Supplementary Table 5) are particularly interesting. Many of them (*phkB*, *hk-8-2*, and *hk-8-3*) together with others (*tcsB*, *hk-8-1*, *phkA*, *nikA*, and *hk-8-4*) also showed AtfA-dependent regulation when the transcriptomes of the control and the *ΔatfA* mutant strains were compared in unstressed cultures [23]. Meanwhile, some members of this gene group, for example, *nikA*, *ypdA*, *tcsA*, and *tcsB*, are important upstream elements of the HogA/SakA signaling pathway in *A. nidulans*, which regulates oxidative stress response via AtfA itself [73, 74]. In their most recent publication, Silva et al. [31] found that MpkC and SakA, which regulate the expressions of *atfA* and *atfB*, also influence the transcriptions of “two-component signal transduction system” genes, which are important for their own activation in *A. fumigatus*. The most “two-component signal transduction system” genes (7 genes) were upregulated under MSB stress suggesting that this positive feedback regulation is particularly important under this type of oxidative stress (Table 2, Supplementary Table 5). Five of these genes were AtfA dependent (Table 2, Supplementary Table 5), which can be one possible reason for why the highest changes observed in the transcriptome were detected under MSB stress (Figure 2(c); [23]).

Assumption 2. AtfA interacts with other elements of the stress signaling network and/or with other transcriptional regulators. These interactions may modify the biological activity of either AtfA or the interacting elements or both. This assumption is essential when we want to explain the stress-type dependence of the action of AtfA (Figures 2(c), 3(e), Table 1, Supplementary Table 2). Importantly, stress-type-dependent regulations by FgAtf1 of the wheat pathogen fungus *F. graminearum* have also been observed in the formation of antioxidative enzymes [25].

Although both the interacting partners of AtfA and the nature of their interactions have remained yet to be elucidated, it is well known that orthologs and paralogs of AtfA can physically interact with other bZIP transcription factors or even with other signal transduction pathway proteins, for example; Atf1 of *S. pombe* forms heterodimer with Pcr1, another pZIP-type transcription factor, and physically interacts with Cid2 poly(A) polymerase, while AtfB of *A. parasiticus* also forms heterodimer with AP-1, another bZIP protein [30, 75–77]. Moreover, it has also been suggested that AtfA may physically interact with AtfB (AN8643) in *A. nidulans* as well [12].

Assumption 3. AtfA (directly or indirectly) hinders the activity of signaling network elements and/or other transcriptional regulators. It is an important assumption when we would like to explain the behavior of coregulated genes. For example, the number of coregulated genes did not change significantly; however, the spectrum of them altered markedly in the absence of AtfA (Figures 2(a), 2(b), and 2(d)).

This nature of AtfA was most obvious under MSB stress where it likely prevented the response of tBOOH stress-specific genes. As a consequence of this AtfA-mediated asymmetric cross-talk between MSB-elicited and tBOOH-elicited stress responses, several AtfA-dependent genes lost their MSB stress responsiveness (883 genes; Figure 2(c), Supplementary Table 2) while several tBOOH stress-responsive genes became MSB stress responsive (312 genes in total; Supplementary Table 2) in the *ΔatfA* mutant. Importantly, crosstalk between stress signaling pathways (cationic stress *versus* oxidative stress) has been delineated at the level of Hog1 MAPK and Cap1 bZIP transcription factor in the opportunistic human pathogen *C. albicans* by Brown et al. [78]. Further research, including interactome studies, is needed to elucidate the possible interacting partners of the bZIP-type transcription factor AtfA under MSB and tBOOH stresses.

## 5. Conclusions

We set up a mechanistic model to explain the effects of *atfA* gene deletion on the transcriptomic changes observed in oxidative stress-exposed vegetative tissues of *A. nidulans*. According to this model, AtfA can modulate significantly the working of the regulatory network under oxidative stress besides activating directly certain oxidative stress response genes. This model is based on the following premises and assumptions: (i) AtfA regulates positively elements of the signaling network, for example, “two-component signal transduction system” genes, which amplify considerably the number and diversity of AtfA-dependent stress response genes, (ii) the AtfA-dependent upregulation of the “two-component signal transduction system” is particularly important under MSB stress and the absence of this positive feedback regulation explains the detected outstanding transcriptional changes caused by the deletion of *atfA*, (iii) atfA interacts with elements of the signaling network, which leads to the stress-specific regulation of stress response genes, and (iv) AtfA (directly or indirectly) prevents the activation of tBOOH-specific genes under MSB stress, which contribute to the prevention of any significant decrease in the number of coregulated genes in the *ΔatfA* mutant. We hope that his model will help us to gain a deeper insight in the background of the AtfA-dependent regulations and help to understand the sometimes contradictory observations in various fungal species.

## Supplementary Material

Supplementary Table 1 - List of primer pairs used in this study. Supplementary Table 2 - Number and stress responsiveness of genes showing altered regulation by deleting *atfA*. Supplementary Table 3 - Gene enrichment analysis of stress responsive genes. Sheet 1 - Control strain (up- and down-regulated gene groups). Sheet 2 - *ΔatfA* mutant strain (up- and down-regulated gene groups). Sheet 3 - AtfA-dependent genes (up- and down-regulated gene groups). AspGD Gene Ontology Term Finder (http://www.aspergillusgenome.org/cgi-bin/GO/goTermFinder) applying default settings and biological process ontology GO terms as well as the FungFun2 package (https://elbe.hki-jena.de/fungifun/fungifun.php), with default settings and FunCat categories were used. Only hits with p-value < 0.05 were taken into consideration during the evaluation process. Supplementary Table 4 - Selected significant shared GO, FunCat and KEGG pathway terms and their stress dependence under MSB, tBOOH or diamide induced stresses. Supplementary Table 5 - Microarray data of genes belonging to selected gene groups. Composition of the gene groups are defined in the Materials and methods section. Microarray data are expressed as log2 R values. R is equal to SI_treated_/SI_untreated_ and SI values stand for the normalized microarray signal intensities.

## Figures and Tables

**Table 1:** Gene enrichment analysis of AtfA-dependent genes.

Analyzed gene group	Significant shared GO and FunCat terms	Stress dependence
*AtfA-dependent upregulated genes*		
	Alpha-amino acid biosynthetic process (GO)	tBOOH
	Degradation of isoleucine, methionine, valine, arginine (FunCat)	tBOOH
	Peroxisomal transport (FunCat)	tBOOH
	Fatty acid metabolic process (GO)	tBOOH
*AtfA-dependent downregulated genes*		
	Mitotic cell cycle (GO)	MSB, tBOOH, diamide
	Mitotic sister chromatid segregation (GO)	MSB, tBOOH, diamide
	Cytokinesis (GO)	MSB, tBOOH
	Ribosome biogenesis (GO)	tBOOH
	Translation (GO)	MSB, tBOOH
	Tricarboxylic acid cycle (FunCat)	MSB
	Aerobic respiration (FunCat)	tBOOH
	Homeostasis of metal ions (Na, K, Ca, etc.) (FunCat)	diamide
	ER to Golgi vesicle-mediated transport (GO)	MSB

The full lists of the significant shared biological process terms are available in Supplementary Table 3.

**Table 2:** 

Gene ID	Gene name	Known/putative function	Stress conditions					
			Control strain			*ΔatfA* mutant		
			MSB	tBOOH	Diamide	MSB	tBOOH	Diamide
*“Antioxidant enzyme” genes*								
AN9339	*catB*	Catalase	1.4 ± 0.9^∗^	2.4 ± 1.2^∗^	1.2 ± 0.8^∗^	2.9 ± 1.0^∗^	3.1 ± 1.2^∗^	1.6 ± 0.8^∗^
AN10220	*ccp1*	Cytochrome c peroxidase	5.3 ± 1.1^∗^	3.0 ± 1.2^∗^	5.1 ± 1.4^∗^	1.8 ± 0.7^∗^	4.7 ± 1.1^∗^	1.7 ± 0.9^∗^
AN0932	*glrA*	Glutathione reductase	4.8 ± 1.4^∗^	1.3 ± 0.8^∗^	3.4 ± 0.8^∗^	1.7 ± 1.1^∗^	1.6 ± 1.1^∗^	1.2 ± 0.7^∗^
AN2846	*gpxA*	Glutathione peroxidase	2.5 ± 1.3^∗^	4.2 ± 2.0^∗^	3.5 ± 1.1^∗^	1.8 ± 1.0^∗^	1.8 ± 0.9^∗^	2.8 ± 1.0^∗^
AN7567		Glutaredoxin	1.3 ± 0.9^∗^	2.5 ± 1.5^∗^	3.5 ± 0.7^∗^	1.1 ± 0.7^∗^	1.6 ± 1.1^∗^	1.9 ± 0.9^∗^
AN5831		Glutathione transferase	5.3 ± 1.4^∗^	3.1 ± 2.0^∗^	2.3 ± 1.6^∗^	3.3 ± 1.5^∗^	2.0 ± 1.0^∗^	1.6 ± 0.9^∗^
AN3581	*trxR*	Thioredoxin reductase	4.5 ± 1.0^∗^	2.9 ± 1.4^∗^	2.8 ± 1.4^∗^	4.1 ± 1.2^∗^	3.1 ± 1.0^∗^	4.9 ± 1.2^∗^
AN8692	*prxA*	Thioredoxin-dependent peroxidase	3.4 ± 1.0^∗^	3.8 ± 0.8^∗^	3.9 ± 1.4^∗^	3.8 ± 1.4^∗^	2.7 ± 0.8^∗^	3.9 ± 0.9^∗^
*“Siderophore biosynthesis” genes*								
AN5823	*sidA*	L-Ornithine N5-monooxygenase	2.4 ± 0.9^∗^	1.2 ± 0.6^∗^	−2.5 ± 1.1^∗^	0.6 ± 0.8	1.2 ± 0.7^∗^	−0.2 ± 0.5
AN8251	*hapX*	bZIP transcription factor	2.1 ± 0.8^∗^	1.2 ± 0.7^∗^	0.8 ± 0.5^∗^	0.6 ± 0.6	1.5 ± 0.6^∗^	0.8 ± 0.5^∗^
*“Iron-sulfur cluster assembly” genes*								
AN10584		Cysteine desulfurase	2.5 ± 0.8^∗^	1.6 ± 0.9^∗^	2.2 ± 1.0^∗^	0.4 ± 0.5	0.8 ± 0.5^∗^	0.2 ± 0.6
AN2508		Cysteine desulfurase	2.0 ± 0.8^∗^	1.3 ± 0.7^∗^	0.1 ± 0.5	0.0 ± 0.6	0.2 ± 0.5	0.4 ± 1.0
AN4655		Iron-sulfur transferase	1.9 ± 0.8^∗^	2.2 ± 1.0^∗^	2.1 ± 0.9^∗^	2.3 ± 0.9^∗^	3.0 ± 0.7^∗^	2.2 ± 1.1^∗^
AN0447		Role in iron-sulfur cluster assembly	3.2 ± 0.8^∗^	0.8 ± 0.6^∗^	3.1 ± 1.1^∗^	0.6 ± 0.7	0.9 ± 0.8^∗^	1.6 ± 0.7^∗^
AN1407		Role in iron-sulfur cluster assembly	2.2 ± 0.7^∗^	1.2 ± 0.9^∗^	2.9 ± 1.1^∗^	0.6 ± 0.8	0.6 ± 0.7	2.6 ± 0.7^∗^
AN2155		Role in iron-sulfur cluster assembly	3.1 ± 0.8^∗^	1.2 ± 0.6^∗^	3.3 ± 1.4^∗^	0.9 ± 0.7^∗^	1.4 ± 0.8^∗^	2.2 ± 0.7^∗^
AN3632		Role in iron-sulfur cluster assembly	2.9 ± 0.8^∗^	2.0 ± 0.9^∗^	0.5 ± 0.9	1.1 ± 0.6^∗^	2.7 ± 1.1^∗^	0.8 ± 0.6
AN5953		Role in iron-sulfur cluster assembly	1.8 ± 0.8^∗^	1.3 ± 0.7^∗^	1.6 ± 1.0^∗^	0.70 ± 1.1	1.3 ± 0.8^∗^	2.6 ± 0.8^∗^
AN8485		Role in iron-sulfur cluster assembly	2.5 ± 1.0^∗^	3.0 ± 1.2^∗^	1.4 ± 0.7^∗^	1.5 ± 0.5^∗^	1.3 ± 0.6^∗^	1.6 ± 0.5^∗^
AN10012		Role in iron-sulfur cluster assembly	3.1 ± 1.0^∗^	0.8 ± 0.6^∗^	1.2 ± 0.5^∗^	0.3 ± 0.5	0.4 ± 0.6	1.4 ± 0.9^∗^
AN11060		Role in iron-sulfur cluster assembly	3.1 ± 0.9^∗^	0.8 ± 0.7^∗^	1.2 ± 0.9^∗^	2.1 ± 1.1^∗^	0.9 ± 0.7^∗^	2.5 ± 0.9^∗^
*“Two-component signal transduction system” genes*								
AN5296	*tcsA*	Histidine kinase	2.7 ± 0.8^∗^	3.1 ± 1.0^∗^	1.9 ± 0.9^∗^	0.6 ± 0.6	1.9 ± 0.8^∗^	−0.1 ± 0.5
AN1800	*tcsB*	Histidine kinase	4.2 ± 1.1^∗^	2.2 ± 0.7^∗^	1.3 ± 0.7^∗^	2.4 ± 1.2^∗^	1.4 ± 0.8^∗^	−0.3 ± 0.7
AN3101	*phkB*	Histidine kinase	1.5 ± 0.8^∗^	−0.5 ± 0.5	−1.3 ± 0.4^∗^	−0.5 ± 0.6	−0.6 ± 0.6	−1.7 ± 0.8^∗^
AN7945	*hk2*	Histidine kinase	4.2 ± 1.1^∗^	−0.4 ± 0.5	−0.1 ± 0.7	0.2 ± 0.7	0.6 ± 0.7	0.4 ± 0.8
AN4113	*hk-8-2*	Histidine kinase	2.6 ± 0.5^∗^	−0.4 ± 0.6	0.4 ± 0.6	−0.3 ± 0.7	−1.2 ± 0.7^∗^	−1.1 ± 0.6^∗^
AN6820	*hk-8-3*	Histidine kinase	2.9 ± 1.0^∗^	−0.9 ± 0.4^∗^	−2.0 ± 1.1^∗^	0.3 ± 0.6	0.4 ± 0.6	0.9 ± 0.7^∗^
AN2363	*hk-8-6*	Histidine kinase	3.5 ± 1.3^∗^	1.8 ± 0.8^∗^	−0.3 ± 0.6	0.6 ± 0.6	0.2 ± 0.7	−0.9 ± 0.5^∗^
*“Nitrate utilization” genes*								
AN1006	*niaD*	Nitrate reductase	−1.0 ± 0.5^∗^	−2.2 ± 1.1^∗^	−4.3 ± 1.2^∗^	0.5 ± 0.6	−2.2 ± 0.9^∗^	−1.9 ± 1.1^∗^
AN1007	*niiA*	Nitrite reductase	−1.5 ± 0.7^∗^	−1.4 ± 0.7^∗^	−3.2 ± 1.0^∗^	0.2 ± 0.6	−1.9 ± 1.0^∗^	−2.1 ± 1.2^∗^
AN1008	*crnA*	Nitrate transporter	−4.8 ± 1.2^∗^	−1.1 ± 0.7^∗^	−2.1 ± 0.9^∗^	0.9 ± 0.8^∗^	−2.4 ± 1.1^∗^	−0.5 ± 0.5
*Other genes*								
AN1168	cch1	Calcium ion transporter	0.1 ± 0.6	0.8 ± 0.7^∗^	−1.3 ± 0.6^∗^	−0.6 ± 0.7	1.3 ± 0.8^∗^	−0.4 ± 0.7
AN1628	enaB	Calcium ion transporter	−1.5 ± 1.1	2.5 ± 1.2^∗^	−1.2 ± 0.7^∗^	0.4 ± 0.6	1.2 ± 0.5^∗^	0.2 ± 0.7
AN4920	pmcB	Calcium ion transporter	0.7 ± 0.8	1.9 ± 0.9	2.1 ± 1.1^∗^	−1.3 ± 0.6^∗^	1.1 ± 0.5^∗^	1.4 ± 0.7^∗^
AN8842	mid1	Calcium ion transporter	0.5 ± 0.7	0.8 ± 0.7^∗^	0.3 ± 0.6	−1.3 ± 0.7^∗^	1.3 ± 0.6^∗^	−0.2 ± 0.6

Relative transcription levels were quantified with the ΔΔCP value. Mean ± S.D. values are presented. The *actA* (AN6542) gene was used as reference gene. ^∗^Significantly differ from zero according to Student's *t*-test (*p* < 0.05, *n* = 4).

**Table 3:** Specific enzyme activities and sterol contents of the cultures.

Cultures	NR (mkat/kg protein)	G6PDH (mkat/kg protein)	GR (mkat/kg protein)	GPx (mkat/kg protein)	Catalase (kat/kg protein)	Sterol content (*μ*g/mg)
Control strain untreated	2.6 ± 0.3	8.0 ± 1	3.8 ± 0.5	0.40 ± 0.04	0.20 ± 0.02	5.8 ± 0.6
Control strain MSB	1.6 ± 0.3^∗^	8.5 ± 1	4.8 ± 0.6^∗^	0.51 ± 0.05^∗^	0.38 ± 0.03^∗^	5.7 ± 0.2
Control strain tBOOH	0.3 ± 0.1^∗^	8.3 ± 0.9	4.4 ± 0.6^∗^	0.57 ± 0.05^∗^	0.40 ± 0.03^∗^	3.3 ± 0.2^∗^
Control strain diamide	0.6 ± 0.1^∗^	7.8 ± 1	4.5 ± 0.5^∗^	0.77 ± 0.08^∗^	0.30 ± 0.03^∗^	7.0 ± 0.7
*ΔatfA* strain untreated	2.8 ± 0.3	7.4 ± 0.9	3.4 ± 0.4	0.33 ± 0.04	0.18 ± 0.02	6.8 ± 0.7
*ΔatfA* strain MSB	3.1 ± 0.4^∗^	8.0 ± 1	4.6 ± 0.5^∗^	0.46 ± 0.05^∗^	0.43 ± 0.04^∗^	5.7 ± 0.4
*ΔatfA* strain tBOOH	0.3 ± 0.1^∗^	7.7 ± 0.8	4.8 ± 0.5^∗^	0.58 ± 0.06^∗^	0.44 ± 0.04^∗^	2.7 ± 0.2^∗^
*ΔatfA* strain diamide	0.7 ± 0.1^∗^	8.1 ± 1.2	4.6 ± 0.4^∗^	0.44 ± 0.05^∗^	0.43 ± 0.04^∗^	7.3 ± 0.3

Mean ± S.D. values are presented. ^∗^Significantly different from the value measured in the appropriate untreated cultures according to Student's *t*-test (*p* < 0.05, *n* = 3).

## Abbreviations

ER: Endoplasmic reticulum
MSB: Menadione sodium bisulfite
tBOOH: *t*-Butylhydroperoxide.
