# Supplementary material for: Transcriptome-Based Modeling Reveals that Oxidative Stress Induces Modulation of the AtfA-Dependent Signaling Networks in Aspergillus nidulans
Source: Int J Genomics. 2017 Jul 9;2017:6923849. doi: 10.1155/2017/6923849 (PMC5523550; doi:10.1155/2017/6923849)
Supplement: Supplementary file 1 — Supplementary Table 1 - List of primer pairs used in this study. Supplementary Table 2 - Number and stress responsiveness of genes showing altered regulation by deleting atfA. Supplementary Table 3 - Gene enrichment analysis of stress responsive genes. Sheet 1 - Control strain (up- and down-regulated gene groups). Sheet 2 - ΔatfA mutant strain (up- and down-regulated gene groups). Sheet 3 - AtfA-dependent genes (up- and down-regulated gene groups). AspGD Gene Ontology Term Finder (http://www.aspergillusgenome.org/cgi-bin/GO/goTermFinder) applying default settings and biological process ontology GO terms as well as the FungFun2 package (https://elbe.hki-jena.de/fungifun/fungifun.php), with default settings and FunCat categories were used. Only hits with p-value < 0.05 were taken into consideration during the evaluation process. Supplementary Table 4 - Selected significant shared GO, FunCat and KEGG pathway terms and their stress dependence under MSB, tBOOH or diamide induced stresses. Supplementary Table 5 - Microarray data of genes belonging to selected gene groups. Composition of the gene groups are defined in the Materials and methods section. Microarray data are expressed as log2 R values. R is equal to SItreated/SIuntreated and SI values stand for the normalized microarray signal intensities. [file 6923849.f1.doc]

**Supplementary Table 1** Primer pairs used in RT-qPCR assays.

AN6542

Forward: 5′-GAAGTCCTACGAACTGCCTGATG-3

Reverse: 5′-AAGAACGCTGGGCTGGAA-3’ (51 ºC)

AN7567

Forward: 5´-GCCTCATCTTGCCTCCATTC-3´

Reverse: 5´-CTTTGTCGCCTCACTGCC-3´

AN2846

Forward: 5´-TTACCAGTCCATCAAAGCCAAG-3´

Reverse: 5´-TTCAGCCAAGTCCAAAGAGG-3´

AN0932

Forward: 5´-CCGTTCGTCTCTCGTCTGTTC-3´

Reverse: 5´-GTCATACTGTTTTGTCTCCACCG-3´

AN5831

Forward: 5´-CTGAGGGGTGAGATTGAGG-3´

Reverse: 5´-AAAGAGATACGGCTGGTGC-3´

AN3581

Forward: 5´-TGGCAGAACGGTATCAGCG-3´

Reverse: 5´-GCGGACAAGCACGGTAAC-3´

AN8692

Forward: 5´-CTGGACTGAGGAGAAGGG-3´

Reverse: 5´-CAAGGACGGCAACAACATCG-3´

AN10220

Forward: 5´-GCGACCAAGAACCAAGACC-3´

Reverse: 5´-AACCAACAGGCGGAAAAACTC-3´

AN0447

Forward: 5´-GACTTCCCTACCTCCTTCTTG-3´

Reverse: 5´-GCTCCACTCTTTTCCACGG-3´

AN10012

Forward: 5´-CTAACACAAGCGGATGAGCC-3´

Reverse: 5´-GGAACAACAAGACGGGAACC-3´

AN10584

Forward: 5´-GGTCTCTGTTTCGCTCTCTG-3´

Reverse: 5´-ACGGTTTGCCTCTTCATCATTC-3´

AN11060

Forward: 5´-CAAGTCGTCAGTCACCCTC-3´

Reverse: 5´-ATTCTCATCTCCACCATCGTC-3´

AN1407

Forward: 5´-CGACGCTCTCTCTGACTAC-3´

Reverse: 5´-CCTATGACTGTGGCTAAACTG-3´

AN2155

Forward: 5´-AGTCCACATTTTCGTCGCTTCTC-3´

Reverse: 5´-TCACCCCAGTCCACATCTTTC-3´

AN2508

Forward: 5´-GGACCAAGGAACACGACC-3´

Reverse: 5´-CGCATCAGCCCCAATCAAG-3´

AN3632

Forward: 5´-GAGAATCCCTACGAAGTCACC-3´

Reverse: 5´-GCTGGCAACGAAAATCCGC-3´

AN4655

Forward: 5´-AGACTTTTGGCTGTGGCTCG-3´

Reverse: 5´-TTGATGGCGGCGGTAACG-3´

AN5953

Forward: 5´-CGTCCAGCAGTGTCATCC-3´

Reverse: 5´-TCCATCGTCATCGGTCCTTG-3´

AN8485

Forward: 5´-CGACACAACCCCAGACTTC-3´

Reverse: 5´-AACAGCCGCATAGACCTC-3´

AN8251

Forward: 5´-CGAAATGGGATGGTGGCG-3´

Reverse: 5´-TCTTGACGAACGAGGCGG-3´

AN5823

Forward: 5´-GCTTCACCTTCCTCAACTAC-3´

Reverse: 5´-ACTTCAATCACCTCCTCTCC-3´

AN1800

Forward: 5´-TCATACAATCCGCCTTACAGC-3´

Reverse: 5´-ATCCCAATCCTTCATCCCC-3´

AN2363

Forward: 5´-GGAAGAAGCCTACAAACACCG-3´

Reverse: 5´-GCAATACGATAGCCGAACAGTC-3´

AN2581

Forward: 5´-TGCTTCCTCAGTCCTCTC-3´

Reverse: 5´-ACAAATCCCGTCCTCCTTAG-3´

AN3101

FORWARD: 5´-TGGTGGCTTTTACGGATTGG-3´

Reverse: 5´-AGTTCTTTCAGGGTCGGC-3´

AN4113

Forward: 5´-TTCTCGCCAGCATCTTCGC-3´

Reverse: 5´-AGCCGTAGTTCGTCGTCAG-3´

AN5296

Forward: 5´-GTTGAGCCGCATCTACCG-3´

Reverse: 5´-CTATCTTTTCCCCGACCACG-3´

AN6820

Forward: 5´-AGGCTGATGGCTTATGGC-3´

Reverse: 5´-CTGGATGGGCACGGAAAC-3´

AN7945

Forward: 5´-CGATGTCACTTACGCCGC-3´

Reverse: 5´-AAACCGCTCTTGCTGCTG-3´

AN1168

Forward: 5´-CAATCCCCTTACAAGACCTTAC-3´

Reverse: 5´-AACCCAGAGACTTCCAATCC-3´

AN1189

Forward: 5´-ACGAGCACCCACGATAAC-3´

Reverse: 5´-CTGACGAAGCATACGAGCG-3´

AN1628

Forward: 5´-TGTTGTCGGGTTCTTCCAGG-3´

Reverse: 5´-AGGGCTTCATCGGTCTCG-3´

AN4920

Forward: 5´-CGGTTGTCTCTCTTTCTTTGGG-3´

Reverse: 5´-GATGATTCGTCGCACTTGATTCC-3´

AN8842

Forward: 5´-CATAAGAGCGATAGTGGTCAAAC-3´

Reverse: 5´-TAACAGTAGTCAGAAGTGCCG-3´

AN9339

Forward: 5’-CCGAGCCCGACAACACTTAC-3’

Reverse: 5’-GTTCAGCGACGACAATGACG-3’

AN1006

Forward: 5’-TATGTCGTCCCAAAACCCG-3’

Reverse: 5’-TTATTCTTCGTCCGCCTCC-3’

AN1007

Forward: 5’-TCGTGATTGGAGAAGAGCC-3’

Reverse: 5’-CGGGTATTGAGGTAGTAGTC-3’

AN1008

Forward: 5´-CGCTTCTTCATCGGCATCC-3´

Reverse: 5´-CATTTTCCAGTCGGGGTGTC-3´
