# Supplementary material for: Transcriptome-Based Modeling Reveals that Oxidative Stress Induces Modulation of the AtfA-Dependent Signaling Networks in Aspergillus nidulans
Source: Int J Genomics. 2017 Jul 9;2017:6923849. doi: 10.1155/2017/6923849 (PMC5523550; doi:10.1155/2017/6923849)
Supplement: Supplementary file 4 [file 6923849.f4.doc]

**Supplementary Table 4** Selected significant shared GO and FunCat terms and their stress dependence under MSB, tBOOH or diamide induced stresses.

| **Control strain** | | | | ***ΔatfA* mutant** | | | |
| --- | --- | --- | --- | --- | --- | --- | --- |
| **Process term** | **Studied stress** | | | **Process term** | **Studied stress** | | |
|  | **MSB** | **tBOOH** | **Diamide** |  | **MSB** | **tBOOH** | **Diamide** |
| oxygen and radical detoxification (FunCat) | Up | Up |  | oxidative stress response (FunCat) | Up |  |  |
| unfolded protein response (FunCat) |  | Up |  | unfolded protein response (FunCat) |  | Up |  |
|  |  |  |  | heat shock response (FunCat) | Up | Up |  |
| metabolism of thioredoxin, glutaredoxin, glutathion (FunCat) | Up |  |  | metabolism of thioredoxin, glutaredoxin, glutathion (FunCat) | Up |  |  |
| glutathione conjugation reaction (FunCat) | Up |  |  |  |  |  |  |
| mitotic cell cycle (GO) | Down |  | Down |  |  |  |  |
| mitotic sister chromatid segregation (GO) | Down | Down | Down |  |  |  |  |
| mitotic nuclear division (GO) | Down | Down | Down |  |  |  |  |
| cytokinesis (GO) | Down | Down |  |  |  |  |  |
| DNA replication (GO) |  |  | Down | DNA-dependent DNA replication (GO) | Down |  |  |

Continued on next page.

Continued from previous page.

| ribosome biogenesis (FunCat) | Down | Down | Down | ribosome biogenesis (FunCat) | Down | Down | Down |
| --- | --- | --- | --- | --- | --- | --- | --- |
| tRNA processing (GO) |  | Down |  | tRNA synthesis (GO) | Down |  |  |
| translation (FunCat) | Down | Down |  | translation (GO) | Down | Down |  |
| biosynthesis of leucine (FunCat) | Up | Up |  | biosynthesis of leucine (FunCat) | Up |  |  |
| degradation of leucine (FunCat) | Up | Up |  | degradation of leucine (FunCat) | Up |  |  |
| biosynthesis of valine (FunCat) | Up |  |  | biosynthesis of valine (FunCat) | Up |  |  |
| degradation of valine (FunCat) | Up | Up |  | degradation of valine (FunCat) | Up |  |  |
|  |  |  |  | biosynthesis of threonine (FunCat) | Up |  |  |
| degradation of threonine (FunCat) |  | Up |  | degradation of threonine (FunCat) | Up |  |  |
|  |  |  |  | biosynthesis of isoleucine (FunCat) | Up |  |  |
| degradation of isoleucine (FunCat) | Up | Up |  | degradation of isoleucine (FunCat) | Up |  |  |
| degradation of serine (FunCat) | Up |  |  |  |  |  |  |
|  |  |  |  | degradation of arginine (FunCat) | Up |  |  |
|  |  |  |  | degradation of proline (FunCat) | Up |  |  |
|  |  |  |  | assimilation of ammonia, metabolism of the glutamate group (FunCat) | Up |  |  |

Continued on next page.

Continued from previous page.

|  |  |  |  | urea catabolism (not urea cycle) (FunCat) | Up |  |  |
| --- | --- | --- | --- | --- | --- | --- | --- |
| protein localization to endoplasmic reticulum (GO) | Down |  |  | non-vesicular ER transport (FunCat) | Down |  |  |
| ER to Golgi vesicle-mediated transport (GO) | Down |  |  |  |  |  |  |
| protein localization to peroxisome (GO) |  | Up |  |  |  |  |  |
| peroxisomal transport (FunCat) |  | Up |  | peroxisomal transport (FunCat) |  | Up |  |
| fatty acid ß-oxidation (GO) |  | Up |  |  |  |  |  |
| drug/toxin transport (FunCat) | Up | Up | Up | drug/toxin transport (FunCat) |  | Up |  |
| iron-sulfur cluster assembly (GO) | Up |  |  |  |  |  |  |
| tricarboxylic-acid pathway (FunCat) | Down |  |  |  |  |  |  |
| homeostasis of metal ions (Na, K, Ca etc.) (FunCat) |  |  | Down | homeostasis of metal ions (Na, K, Ca etc.) (FunCat) |  | Up |  |

The full lists of the significant shared biological process terms are available in Supplementary Table 3.
